# The Effect of GPX2 on the Prognosis of Lung Adenocarcinoma Diagnosis and Proliferation, Migration, and Epithelial Mesenchymal Transition

**DOI:** 10.1155/2022/7379157

**Published:** 2022-07-18

**Authors:** Yu-peng Li, Rui Lin, Ming-zhu Chang, Yi-jiu Ai, Si-ping Ye, Hui-ming Han, Yao-yue Zhang, Han Mou, Run-hong Mu, Xiao Guo

**Affiliations:** ^1^Basic Medical College of Beihua University, Jilin 132013, China; ^2^Pharmacy College of Beihua University, Jilin 132013, China; ^3^Affiliated Hospital of Beihua University, Jilin 132000, China; ^4^Stomatology College of Beihua University, Jilin 132013, China; ^5^Laboratory Medical College of Beihua University, Jilin 132013, China

## Abstract

**Objective:**

To investigate the expression of glutathione peroxidase 2 (GPX2) in human lung adenocarcinoma tissues and its effect on the biological function of lung adenocarcinoma A549 cells.

**Methods:**

The expression of GPX2 in lung adenocarcinoma and its effect on survival were analyzed by the TCGA database and the GEPIA 2 database. A total of 45 cases of primary lung adenocarcinoma tissue specimens and 45 cases of their paracancerous tissue specimens were collected, and the expression of GPX2 in the two types of tissues was detected by immunohistochemistry. Lung adenocarcinoma A549 cells were divided into the GPX2 overexpression group (GPX2), the GPX2 knockdown group (si-GPX2), the empty vector group (Vector), the siRNA negative control group (si-NC), and the WT group; the mRNA level and protein expression of GPX2 in each group of A549 cells were detected by real-time fluorescence quantitative PCR and Western blotting; the proliferation activity of each group of cells was detected by the CCK-8 assay; the effect of GPX2 on cell migration and invasion ability was detected by the scratch assay and the Transwell invasion assay; the apoptosis of each group of cells was detected by flow cytometry; Western blotting was performed to detect the expression levels of Bax, Bcl-2, E-cadherin, vimentin, and MMP2 and MMP9 proteins in each group of cells.

**Results:**

Bioinformatics analysis showed that the expression of GPX2 was strongly correlated with the prognosis of lung adenocarcinoma patients (*P* < 0.01). The positive expression rates of GPX2 in lung adenocarcinoma and its paracancerous tissues were 66.0% and 15.7%, respectively (*P* < 0.05). The results of RT-qPCR and Western blotting showed that the expression level of GPX2 mRNA and protein in A549 cells in the GPX2 group increased, which was significantly higher than that in the WT group (*P* < 0.05); the expression levels of GPX2 mRNA and protein in A549 cells in the si-GPX2 group were the same, that is, significantly lower than the WT group (*P* < 0.05). GPX2 overexpression promoted the proliferation, migration, and invasion of A549 cells and inhibited their apoptosis; the results in the si-GPX2 group were opposite to those in the GPX2 group. Compared with the WT group, the expression of Bcl-2, vimentin, and MMP2 and MMP9 protein in the GPX2 group increased (*P* < 0.05), while the expression of Bax and E-cadherin protein decreased in the GPX2 group (*P* < 0.05); the results in the si-GPX2 group were opposite to those in the GPX2 group.

**Conclusion:**

The expression of GPX2 in lung adenocarcinoma is related to the prognosis of patients. It is proved that GPX2 can promote the migration and invasion of lung adenocarcinoma cells and is related to the EMT/*β*-catenin pathway. Thus, GPX2 is expected to be an important target for the diagnosis and treatment of lung adenocarcinoma.

## 1. Introduction

Lung cancer has a perennial high incidence and mortality rate worldwide [1]. Most lung cancers are detected at an advanced stage, resulting in a short survival period for patients [2]. Non-small cell lung cancer is the most common type of lung cancer and can be further divided into lung adenocarcinoma, lung squamous cell carcinoma, and lung large cell carcinoma. Lung adenocarcinoma accounts for 45% to 50% of non-small cell lung cancer, and its incidence is increasing year by year, posing a serious risk to human health [3]. The occurrence and development of lung cancer is closely related to abnormal gene expression and regulation. The predominant options for prolonging the survival and improving the quality of life of lung cancer patients are now still mainly chemotherapy and radiotherapy [4]. Although great progress has been made in molecularly targeted therapy worldwide in recent years, the average 5-year survival rate of patients with non-small cell lung cancer is still as low as 15% [5]. The pathogenesis of lung adenocarcinoma, such as epigenetic regulation and alterations at the gene and protein levels, has not yet been fully elucidated. Therefore, it is of great significance to explore the molecular mechanism of lung adenocarcinoma in order to better understand the occurrence, development, and treatment of lung adenocarcinoma.

Reactive oxygen species (ROS) are the products of redox reactions in life and are involved in various biological processes such as signal transduction, gene expression, cell proliferation, differentiation, and apoptosis [6]. Excessive ROS can lead to oxidative stress in the body, which in turn leads to diseases such as aging, cardiovascular, and cerebrovascular diseases, and cancer [7]. Glutathione peroxidase (GPX) is a key antioxidant enzyme, which can eliminate the damage caused by ROS and maintain the metabolic balance of ROS in the body. GPX2 is a member of the GPX family [8] and is mainly expressed in the gastrointestinal system, but also in epithelial cells of the esophagus, lung, and liver [9]. Numerous studies have confirmed that the expression of GPX2 is significantly upregulated in gastrointestinal tumors, liver tumors, glioblastoma, lung adenocarcinoma, breast cancer, and other cells [10–15]. Du et al. [16] used the online tools GEPIA and cBioPortal database analysis to confirm that the high expression of GPX2 mRNA is related to the survival rate of patients with lung adenocarcinoma, but the mechanism research still needs to be improved. Gao et al. [17] found that GPX2 expression has a significant effect on the prognosis of patients with lung adenocarcinoma by using Oncomine and Kaplan–Meier plotter databases. In recent years, the reports on the molecular mechanism and function of GPX2 have provided broad ideas to study the mechanism and treatment of human-related diseases.

In this study, the expression of GPX2 in lung adenocarcinoma and its effect on the prognosis of lung adenocarcinoma were analyzed by bioinformatics, then detected the expression level of GPX2 in lung adenocarcinoma tissues by immunohistochemistry, and observed the effect of GPX2 on the phenotype of A549 cells by overexpression and knockdown of GPX2 gene in lung adenocarcinoma A549 cells, aiming to clarify the role of GPX2 in the development of lung adenocarcinoma and provide theoretical basis for the diagnosis and treatment of lung adenocarcinoma.

## 2. Materials and Methods

### 2.1. Main Reagents

DMEM (HyClone, USA), Fetal Bovine Serum (Gibco, USA), Cell Counting Kit 8 (Beyotime Biotechnology, China), PrimeScript™ RT Reagent Kit and SYBR PremixExTaq™ II Reagent Kit (TaKaRa, Japan), RIPA Lysis Buffer (Beyotime Biotechnology, China), Lipofectamine® 2000 (Invitrogen, USA), AnnexinV-FITC/7-AAD Reagent kit (Sino Biological, China), Bax, Bcl-2, E-cadherin, vimentin antibodies, MMP2 and MMP9 antibodies (Abcam, UK), electrochemiluminescence reagents (Azure Biosystems, USA), Transwell chamber (Corning-Costar, USA). TRIzol reagent and primers for PCR were purchased from Shanghai Biotech Bioengineering Co., Ltd. GPX2-siRNA and PIRES-EGFP-GPX2 were synthesized by Shanghai Sangon Bioengineering Co., Ltd.

### 2.2. Source of Specimens

During the period of March 2019 and December 2020, 45 cases of primary lung adenocarcinoma tissue specimens and 45 cases of their paracancerous tissue specimens were collected in the Department of Pathology at Beihua University's Affiliated Hospital of Pathology. A total of 28 cases were male and 17 cases were female, aged 42–75 years, with a mean age of 54.2 years. None of the patients received radiotherapy or chemotherapy before surgery, and the diagnosis was further confirmed following surgery by pathological testing.

### 2.3. RNA Sequencing Data Mining and Statistical Analysis

For GPX2 gene expression analysis in lung adenocarcinomas, tumor RNA-seq and mRNA expression data from tumor-paired normal tissue samples were downloaded from The Cancer Genome Atlas (TCGA) database (https://portal.gdc.cancer.gov/) and Gene Expression Profiling Interactive Analysis 2 (GEPIA 2) database (https://gepia2.cancer-pku.cn/#index). The Kaplan–Meier method was used to determine the association between GPX2 and patient survival. Log-rank was used to test KM survival analysis and to compare survival differences between two or more groups. Time ROC analysis was used to compare the prediction accuracy and risk score of the GPX2 gene. Cox regression models were performed for univariate and multivariate analysis. All statistical analyses were performed using GraphPad Prism 7.

### 2.4. Cell Culture and Transfection

Human lung adenocarcinoma A549 cell line was preserved by the laboratory of the School of Pharmacy, Beihua University. A549 cells were cultured in the DMEM containing 10% FBS in a 37°C, 5% CO_2_ incubator. A549 cells in logarithmic growth phase were inoculated into a 24-well plate (1.0 × 10^5^ cells per well). When the cells were completely adherent and the cell density reached 70–80%, transfection was carried out according to the instructions of Lipofectamine®2000 kit. The experiments were divided into the overexpression PIRES-EGFP-GPX2 group (GPX2), empty vector group (Vector), siRNA GPX2 knockdown group (si-GPX2), siRNA negative control group (si-NC), and WT group. The treated transfection reagent was added to the culture plate and placed in the cell incubator, and the subsequent experiments were carried out 48 h after transfection.

### 2.5. Immunohistochemistry and Determination Criteria

The paraffin-embedded specimens were made into serial paraffin sections with a thickness of 5 *μ*m, mounted on slides, and baked at 60°C for 24 h. After the sections were cooled, the steps of deparaffinization, hydration, and antigen thermal repair were performed with reference to the instructions of the immunohistochemical staining kit. 1 : 1000 dilution of mouse anti-human monoclonal antibody GPX2 was incubated overnight at 4°C, and then, the secondary antibody for 30 min at room temperature was incubated; DAB was used for color development. The staining results were determined by double-blind trials using phosphate buffer as a negative control, and the cells were considered positive if the cell pulp or nucleus showed brownish-yellow granules. Protein expression was scored according to the staining intensity and the number of positive cells, with the following criteria: (1) the number of positive cells 0–5% was scored as 0, 6%–25% was scored as 1, 26%–50% was scored as 2, and >50% was scored as 3; and (2) the positive intensity was scored as 0 for colorless, 1 for pale yellow, 2 for brownish yellow, and 3 for brownish brown. The points of (1) (2) were added together, and the total score was 0 as negative, 1–2 as weakly positive, 3–4 as moderately strong positive, and 5–6 as strongly positive.

### 2.6. Detection of the Expression of GPX2 mRNA by Real-Time qPCR (RT-qPCR)

Total RNA was extracted with TRIzol reagent, and the concentration and purity of RNA were calculated by measuring the absorbance (*A*) values at 260 nm and 280 nm with a UV spectrophotometer. The total RNA was reverse transcribed into cDNA according to the instructions of the Takara reverse transcription kit. The obtained cDNA was used as a template and SYBR Premix Ex Taq was used as a fluorochrome for RT-qPCR detection. Primers were designed using Primer 5.0 software, GPX2-F: 5′-CAAGACTTGG-3′; GPX2-R: 5′-GGTCTCTTC-3′; *β*-actin-F: 5′-GATGAGATTGGCATGGCTTT-3′; *β*-actin-R: 5′-CACCTTCACCGTTCCAGTTT-3′. *β*-Actin was used as an internal reference. RT-qPCR reaction conditions were as follows: 95°C for 15 min; 95°C for 10 s, 60°C for 30 s, 72°C for 30 s, 40 cycles; 60°C for 30 s, 95°C for 45 s, and 20°C for 10 min. The GPX2 mRNA expression level in each sample was calculated and analyzed using the 2^−ΔΔCt^ formula. All experiments were performed at least thrice.

### 2.7. Detection of Cell Proliferation Activity by CCK-8 Assay

Cells in each group were evenly plated into 96-well plates at 2 × 10^3^ cells/well (100 *μ*L/well), and each group had 3 parallel wells. After 24 h, 48 h, 72 h, and 96 h, 90 *μ*L culture medium and 10 *μ*L CCK-8 reagent were added and blank control wells containing only culture medium and CCK-8 reagent were set. After culturing in the 5% CO_2_ incubator at 37°C for 2 h, the absorbance (*A*) value of the cells was detected at 450 nm. GraphPad Prism 7 software was used to draw the proliferation curve of each group of cells over time. All experiments were performed at least thrice.

### 2.8. Detection of Cell Migration by Scratch Test

After the cells in each group were cultured overnight in 6-well plates (1 × 10^6^ cells/well), scratches were carried out vertically with a 200-*μ*L pipette tip to keep the scratches in each well basically the same; the cells were washed three times with PBS and were continued to be cultured in a serum-free medium in the 5% CO_2_ incubator at 37°C. The cell distribution status was observed after 24 h and 48 h. Microscopic photographs were taken, and the ratio of the scratch area to the picture area was calculated to determine the cell migration rate. All experiments were performed at least thrice.

### 2.9. Detection of Cell Invasion by Transwell Assay

After 48 h of transfection, the cells in each group were digested with trypsin, and the concentration of the cell suspension was adjusted to 2 × 10^5^ cells/mL. 100 *μ*L of the cell suspension was added into the Transwell upper chamber, and 600 *μ*L of the complete medium was added into the lower chamber. 100 *μ*L of the DMEM was added to the upper chamber of the control group. After being cultured in the incubator for 24 h and 48 h, the upper chamber was taken out and the liquid in the lower chamber was discarded. The cells were stained with Wright's-Giemsa Staining Solution for 90 s, rinsed with PBS, air-dried, and placed under an inverted microscope for counting and taking pictures. All experiments were performed at least thrice.

### 2.10. Detection of Apoptosis with a Flow Cytometer

The cells in each group were digested with trypsin 48 h after transfection, and the suspension concentration was adjusted to 1 × 10^5^ cells/group. FlowJo V10 was used to analyze and plot the data after the cells were treated according to the instructions of the apoptosis kit. All experiments were performed at least thrice.

### 2.11. Detection of the Expression of Proteins by Western Blot

In order to determine the total protein concentration of each group by BCA assay, RIPA lysis was used to extract proteins from GPX2 overexpression, empty vector, and knockdown groups. Samples containing the same amount of protein (30 *μ*g) were separated using 10% sodium dodecyl sulfate polyacrylamide gel electrophoresis (SDS-PAGE). Subsequently, the proteins were transfer embedded onto polyvinylidene fluoride (PVDF) membranes, and blocked with 5% skim milk at room temperature for 2 h, and then, the PVDF membranes with primary antibody diluent (1 : 2000) were incubated overnight at 4°C. After washing the membranes three times with TBST, 10 minutes per time, they were incubated with the secondary antibody at 37°C for 1 h. After washing, the bands were developed and exposed with ECL chemiluminescent solution. Gray value analysis of the bands was performed using ImageJ 1.8.0 software. Using GADPH as an internal reference, the protein expression levels of GPX2, Bax, Bcl-2, E-cadherin, vimentin, and MMP2 and MMP9 were calculated. All experiments were performed at least thrice.

### 2.12. Statistical Analysis

Statistical analysis was performed using SPSS 19.0 (SPSS Inc. USA) statistical software. The cell proliferation activity and protein expression level in cells were in accordance with normal distribution, and the cell migration rate and the number of invasive cells were expressed as $\bar{x}\pm s$. The comparison between groups was performed by one-way ANOVA. *P* < 0.05 indicated a value with a statistical significance.

## 3. Results

### 3.1. Analysis of GPX2 Expression in Lung Adenocarcinoma

**Figure 1:**
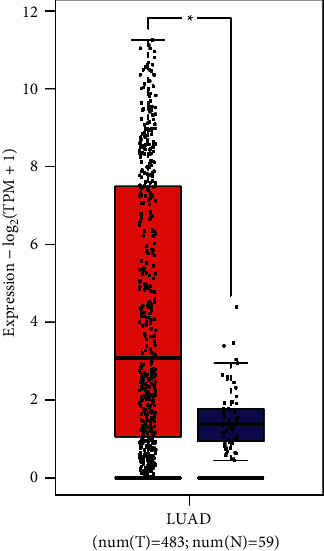
TCGA and GEPIA analysis of GPX2 expression in normal tissues and lung adenocarcinoma (^∗^*P* < 0.01).

Figure 1 depicts the distribution of GPX2 gene expression in tumor tissues and normal tissues. By searching the expression of GPX2 in lung adenocarcinoma in GEPIA 2 database and matching with TCGA normal data, it revealed that GPX2 expression levels were significantly higher in lung adenocarcinoma tissues than in normal tissues, and there was a highly significant difference in GPX2 expression levels between the two groups of samples (*P* < 0.01).

### 3.2. Effect of GPX2 on the Prognosis of Lung Adenocarcinoma

**Figure 2:**
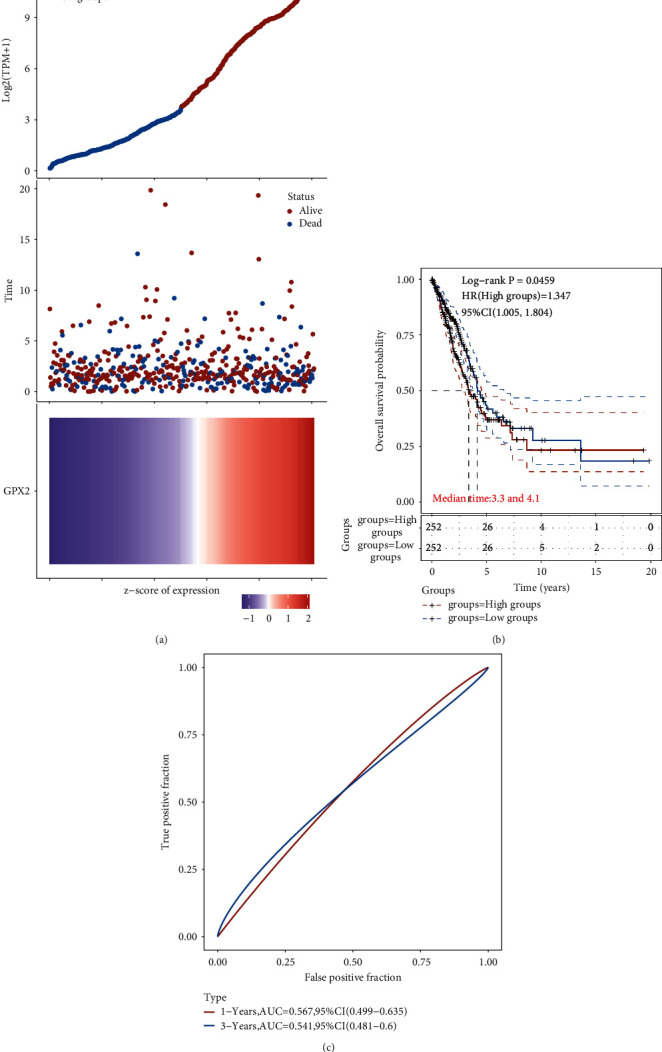
GPX2 expression and survival status. (a) GPX2 expression, survival time, and survival status in TCGA database; (b) KM survival curve distribution of GPX2 gene in TCGA database; (c) ROC curve and AUC values of GPX2 at different times.

The relationship between GPX2 gene expression and lung adenocarcinoma was analyzed by the GEPIA database. As shown in Figure 2(a), GPX2 gene expression was significantly associated with prognosis. The risk curve and scatter plot illustrate the relationship between the risk score and the corresponding survival status of lung adenocarcinoma patients. The higher the risk score, the higher the mortality. The results of the distribution of KM survival curves showed that the median survival time (MS) of the GPX2 high expression group (MS = 3.3) was significantly lower than that of the GPX2 low expression group (MS = 4.1), and the overall survival rate (OS) of patients with high GPX2 expression was significantly lower than that in the low GPX2 expression group (*P*=0.0459, Figure 2(b)) suggesting that high GPX2 expression is associated with poor prognosis in patients with lung adenocarcinoma. In order to evaluate the specificity and sensitivity of the risk score in predicting the prognosis of lung adenocarcinoma patients, the area under the ROC curve value (AUC) of the risk score was evaluated. The AUC value of risk score at 1 and 3 years was 0.567 and 0.541, respectively (Figure 2(c)).

### 3.3. Expression of GPX2 in Lung Adenocarcinoma and Paraneoplastic Tissues

**Figure 3:**
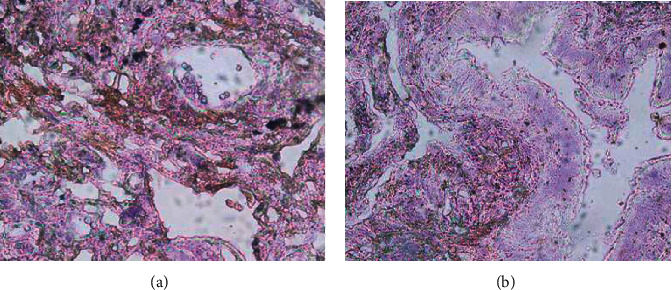
The expression of GPX2 in lung adenocarcinoma and paracancerous tissues via IHC (×200). (a) Lung adenocarcinoma tissues; (b) paracancerous tissues.

IHC showed that GPX2 was highly expressed in lung adenocarcinoma tissues and lowly expressed in paracancerous tissues (Figure 3). The positive expression rates of GPX2 in lung adenocarcinoma tissues and paracancerous tissues were 60.0% and 15.5%, respectively, with statistically significant differences between groups *P* < 0.05 (Table 1).The comparison between groups was performed by the chi square test.

### 3.4. GPX2 mRNA and Protein Expression Levels in A549 Cells in Each Group after Transfection

**Figure 4:**
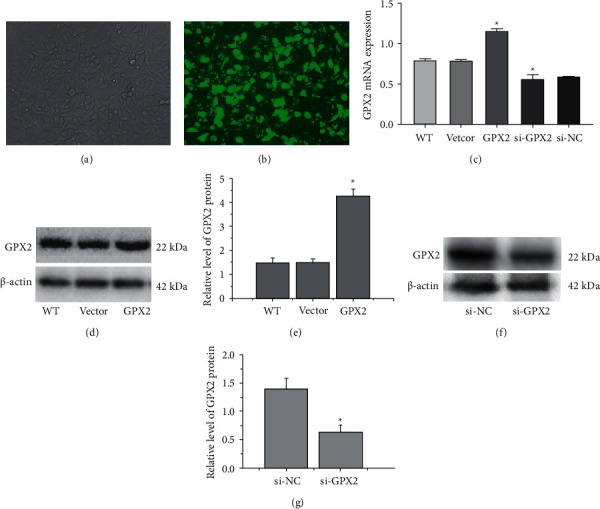
(a) Image of A549 cells at normal light (×100). (b) Image of A549 cells at fluorescent. (c) The expression level of GPX2 in A549 cells in each group. (d, e) The expression level of GPX2 in A549 cells transduced with GPX2 overexpression vector. (f, g) The expression level of GPX2 in A549 cells transduced with GPX2 siRNA (^∗^*P* < 0.05).

The transfection of cells in the GPX2 group observed under a fluorescence microscope is shown in Figures 4(a) and 4(b). RT-qPCR results (Figure 4(c)) showed that compared with the WT group and the vector group, the GPX2 mRNA expression in A549 cells in the GPX2 group was increased, and the expression level in the si-GPX2 group was significantly decreased (*P* < 0.05). Western blot results showed (Figures 4(d)–4(g)) that the relative expression level of GPX2 protein in A549 cells in the si-GPX2 group was 0.69 ± 0.15, which was significantly lower than that in the empty vector group (1.58 ± 0.16), and the difference between groups was statistically significant (*P* < 0.05). The relative expression level of GPX2 protein in A549 cells in the GPX2 group was 2.09 ± 0.22, which was significantly higher than that in the vector group (1.58 ± 0.16) and the WT group (1.49 ± 0.18).

### 3.5. Detection of Proliferation Activity of A549 Cells in Each Group by the CCK-8 Method after Transfection

**Figure 5:**
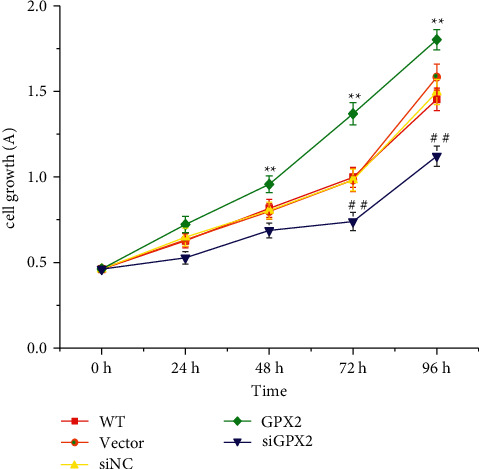
Proliferation activity of A549 cells in each group. ^∗^GPX2 group compared to the WT group and the vector group (*P* < 0.05); ^#^si-GPX2 group compared to the WT group and the si-NC group (*P* < 0.05).

It can be seen from Figure 5 that the proliferation activity of A549 cells was significantly reduced after GPX2 was knocked down. After 48 h and 72 h, the proliferation activity of the si-GPX2 group was lower than that of the WT group and the si-NC group (*P* < 0.05), and the proliferation activity of the GPX2 group was significantly higher than that of the WT group and the vector group (*P* < 0.05).

### 3.6. Effects of GPX2 on the Ability of Cell Migration and Invasion in Each Group

**Figure 6:**
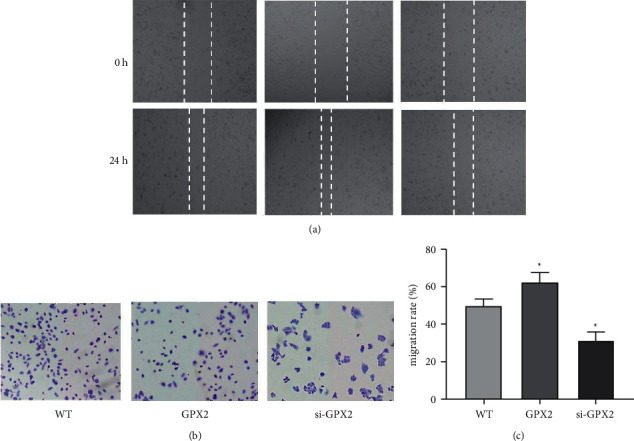
The effect of GPX2 on the migration and invasion ability of A549 cells (×200). (a) The effect of GPX2 on the migration rate of A549 cells; (b) the effect of GPX2 on the invasion of A549 cells; (c) the wound healing rate of cells in each group. ^∗^Compared with the WT group, *P* < 0.01.

As shown in Figure 6(a) and 6(c), the wound healing rate of cells in the si-GPX2 group (31.3% ± 4.6%) was significantly lower (*P* < 0.01) compared to the WT group (50.4% ± 3.7%), while the wound healing rate of cells in the GPX2 group (63.1% ± 4.9%) was significantly higher (*P* < 0.01). The results showed (Figure 6(b)) that GPX2 group had significantly higher cell invasion ability compared to the WT group (*P* < 0.01), and the si-GPX2 group had decreased invasion ability compared to the WT group, and the difference between groups was statistically significant (*P* < 0.05).

### 3.7. Effects of GPX2 on Apoptosis of A549 Cells

**Figure 7:**
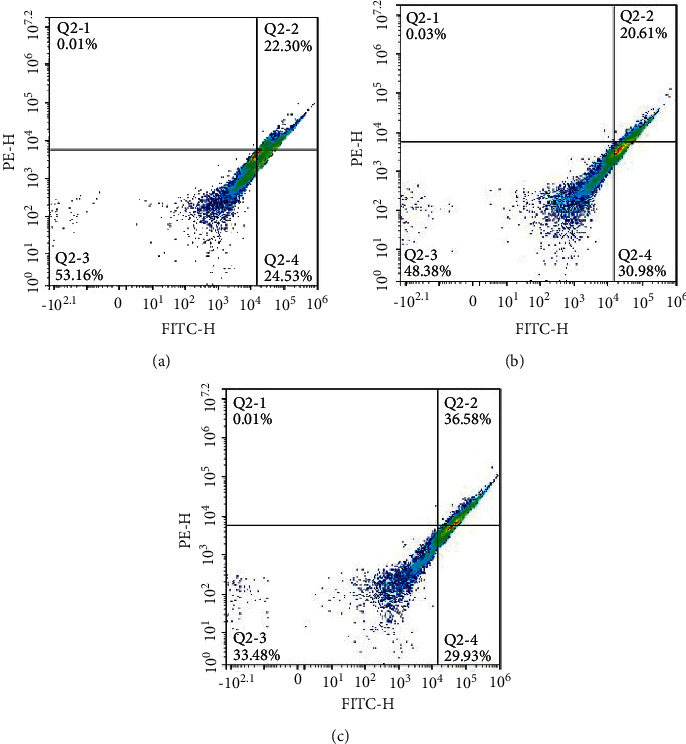
Apoptosis rate of A549 cells in each group: (a) GPX2; (b) WT; (c) si-GPX2.

The results of flow cytometry (Figure 7) showed that compared with the WT group (51.59% ± 4.28%), the apoptosis rate of the GPX2 group (46.83% ± 3.51%) was significantly lower, and the apoptosis rate of the si-GPX2 group (66.51% ± 4.94%) was significantly increased (*P* < 0.05).

### 3.8. Expression Levels of Bax, Bcl-2, E-Cadherin, Vimentin, and MMP2 and MMP9 Proteins in Each Group of Cells

**Figure 8:**
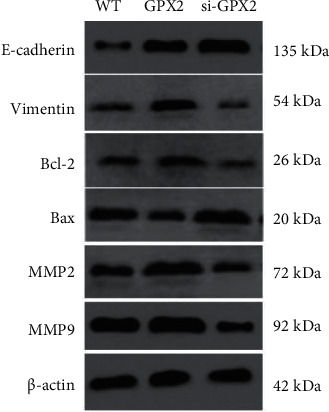
Expression levels of each protein in each group of cells.

The expression level of E-cadherin protein (Figure 8) in the si-GPX2 group (2.137 ± 0.013) was significantly higher than that in the WT group (1.000 ± 0.082) (*P* < 0.05), and the GPX2 group (0.921 ± 0.011) was lower than the WT group (1.000 ± 0.082) (*P* < 0.05). The expression level of vimentin protein in the si-GPX2 group (0.370 ± 0.006) was significantly lower than that in the WT group (1.000 ± 0.049), and the GPX2 group (0.931 ± 0.008) was significantly higher than that in the WT group (1.000 ± 0.072) (*P* < 0.05). Compared with the WT group, the expression level of Bcl-2 protein in the GPX2 group was increased, the protein level of Bax was decreased, and the protein expression levels of MMP2 and MMP9 were significantly upregulated (*P* < 0.05).

## 4. Discussion

Tumorigenesis is an extremely complex process that includes genetics, somatic aberrations, and copy number variants [18]. Only by better understanding the changing pattern of cancer can we diagnose, treat, and prevent it accurately. In recent years, big data bioinformatics analysis methods have entered our vision and gradually become a new means to decipher the pathogenic mechanism of tumors and to ensure the molecular biology analysis of cancer [19].

GPX family plays an important role in the antioxidant process, not only scavenging hydrogen peroxide and lipid peroxides, but also playing an important role in cancer progression [20]. In this study, we analyzed the expression and prognosis of GPX2 in lung adenocarcinoma based on bioinformatics and found that the expression level of GPX2 was significantly higher in lung adenocarcinoma tissues compared with normal lung tissues, which initially confirmed that reduced GPX2 expression was associated with the prognosis of lung adenocarcinoma. To further determine the effect of GPX2 gene on the growth and metastasis process of lung adenocarcinoma, GPX2 expression in clinical lung adenocarcinoma tissues compared with paraneoplastic tissues was detected by immunohistochemistry, and the results showed that GPX2 expression level was significantly increased in lung adenocarcinoma tissues. Overexpression and knockdown of GPX2 gene in A549 cells showed significantly enhanced proliferative activity, decreased apoptosis, and enhanced migration and invasiveness of A549 cells in the GPX2 group compared to the WT group, with the opposite results in the si-GPX2 group. This shows that GPX2 plays an important role in the development and progression of lung adenocarcinoma, which is expected to provide a basis for clinical diagnosis and treatment of lung adenocarcinoma.

Yan and Chen [21] demonstrated that the overexpression of GPX2 could reduce oxidative stress-induced apoptosis in MCF7 cells, while the knockdown of GPX2 using RNAi enabled MCF7 cells to significantly increase their sensitivity to oxidative stress-induced apoptosis. It was found that A549 cells were more sensitive to apoptosis after the knockdown of GPX2, and an increase in the expression of the pro-apoptotic protein Bax and a decrease in the expression of the anti-apoptotic protein Bcl-2 were also found, which led to the speculation that GPX2 may affect apoptosis by regulating the Bax/Bcl-2 protein family.

Invasion is an important feature of malignant tumors [22–24], and MMP2 and MMP9 are the most widely studied matrix metalloproteinases that play important roles in tumor cell invasion and metastasis. Ning et al. reported [25] that increased expression of MMP2 promoted invasion and migration of laryngeal cancer cells. This led us to further speculate that GPX2 may enhance the invasive ability of lung adenocarcinoma A549 cells by controlling the expression of MMP2 and MMP9 proteins in lung adenocarcinoma cells. Overexpression of GPX2 in A549 cells revealed a significant increase in the expression levels of MMP2 and MMP9 in the cells, while the inhibition of GPX2 expression significantly decreased the expression levels of MMP2 and MMP9 in A549 cells, tentatively confirming that the effect of GPX2 on invasive metastasis of lung adenocarcinoma A549 cells was associated with reduced secretion of MMPs.

Epithelial-to-mesenchymal transition (EMT), as a mesenchymal cell transformation process, is regulated by a variety of factors in different regulatory pathways that lead to the disappearance of epithelial cell markers, and thus, cells acquire mesenchymal properties [12, 26, 27]. Bao et al. [28] found that the expression level of E-cadherin protein was significantly reduced in hepatocellular carcinoma tissues, and vimentin was overexpressed in a variety of malignant tumors of epithelial origin such as breast, gastric, and prostate cancers, involved in the complex process of tumor metastasis and played a role in the migration, adhesion, and epithelial mesenchymal transition of tumor cells and endothelial cells and tumor cell apoptosis [29, 30]. By detecting E-cadherin and vimentin, important molecules related to EMT in A549 cell lines after transfection, we found that the knockdown of GPX2 could activate E-cadherin and inhibit the expression of vimentin to suppress the EMT process in A549 cells, but the specific mechanism of action of GPX2 in regulating EMT needs further in-depth study.

In summary, this study shows that GPX2, a key oncogene, plays an important role in cell proliferation, migration, invasion, and apoptosis, providing a potential target for new therapeutic strategies to prevent and control the development of lung adenocarcinoma, and the molecular mechanism of GPX2 in the occurrence and progression of lung adenocarcinoma still needs to be further explored.

## Figures and Tables

**Table 1:** Expression of GPX2 in lung adenocarcinoma and paracancerous tissues.

Group	Number of patients	Positive	Negatives	Positive rate (%)
Lung adenocarcinoma tissues	45	27	18	60.0
Paracancerous tissues	45	7	38	15.5^∗^

^∗^Compared with the positive rate of the lung adenocarcinoma tissue, *P* < 0.05.

## Data Availability

The databases generated during the current study are available from the corresponding author on reasonable request.

## References

[1] Ferlay J., Parkin D. M., Steliarova-Foucher E. (2010). Estimates of cancer incidence and mortality in Europe in 2008. *European Journal of Cancer*.

[2] Chen W., Zheng R., Baade P. D. (2016). Cancer statistics in China, 2015. *CA: A Cancer Journal for Clinicians*.

[3] Miller K. D., Goding Sauer A., Ortiz A. P. (2018). Cancer statistics for hispanics/latinos, 2018. *CA: A Cancer Journal for Clinicians*.

[4] Zoidis E., Seremelis I., Kontopoulos N., Danezis G. (2018). Selenium-dependent antioxidant enzymes: actions and properties of selenoproteins. *Antioxidants*.

[5] Kipp A. P. (2017). Selenium-dependent glutathione peroxidases during tumor development. *Advances in Cancer Research*.

[6] Seifried H. E., Anderson D. E., Fisher E. I., Milner J. A. (2007). A review of the interaction among dietary antioxidants and reactive oxygen species. *The Journal of Nutritional Biochemistry*.

[7] Haigis M. C., Yankner B. A. (2010). The aging stress response. *Molecular Cell*.

[8] Brigelius-Flohe R., Kipp A. P. (2012). Physiological functions of GPx2 and its role in inflammation-triggered carcinogenesis. *Annals of the New York Academy of Sciences*.

[9] Lei Z., Tian D., Zhang C., Zhao S., Su M. (2016). Clinicopathological and prognostic significance of GPX2 protein expression in esophageal squamous cell carcinoma. *BMC Cancer*.

[10] Liu C., He X., Wu X., Wang Z., Zuo W., Hu G. (2017). Clinicopathological and prognostic significance of GPx2 protein expression in nasopharyngeal carcinoma. *Cancer Biomarkers*.

[11] Naiki-Ito A., Asamoto M., Hokaiwado N. (2007). Gpx2 is an overexpressed gene in rat breast cancers induced by three different chemical carcinogens. *Cancer Research*.

[12] Suzuki S., Pitchakarn P., Ogawa K. (2013). Expression of glutathione peroxidase 2 is associated with not only early hepatocarcinogenesis but also late stage metastasis. *Toxicology*.

[13] Woenckhaus M., Klein-Hitpass L., Grepmeier U. (2006). Smoking and cancer-related gene expression in bronchial epithelium and non-small-cell lung cancers. *The Journal of Pathology*.

[14] Xipell E., Gonzalez-Huarriz M., De Irujo J. J. M. (2016). Salinomycin induced ROS results in abortive autophagy and leads to regulated necrosis in glioblastoma. *Oncotarget*.

[15] Zhang Z., Duan Q., Zhao H. (2016). Gemcitabine treatment promotes pancreatic cancer stemness through the Nox/ROS/NF-*κ*B/STAT3 signaling cascade. *Cancer Letters*.

[16] Du H., Chen B., Jiao N. L., Liu Y. H., Sun S. Y., Zhang Y. W. (2020). Elevated glutathione peroxidase 2 expression promotes cisplatin resistance in lung adenocarcinoma. *Oxidative Medicine and Cellular Longevity*.

[17] Gao H., Zhang B., Pang J., Wang L. (2021). The expression and clinical significance of GPX2 in non-small cell lung cancer were analyzed based on oncomine and kaplan meier plotter databases. *Modern Oncology*.

[18] Liu T., Kan X. F., Ma C. (2017). GPX2 overexpression indicates poor prognosis in patients with hepatocellular carcinoma. *Tumor Biology*.

[19] Hanahan D., Weinberg R. A. (2000). The hallmarks of cancer. *Cell*.

[20] Wang X. F., Zhao H., Zhang G. Y. (2018). Expression and clinical significance of ERO1L in lung adenocarcinoma through data mining. *Journal of Clinical Pulmonary Medicine*.

[21] Yan W., Chen X. (2006). GPX2, a direct target of p63, inhibits oxidative stress-induced apoptosis in a p53-dependent manner. *Journal of Biological Chemistry*.

[22] Aparna M., Rao L., Kunhikatta V., Radhakrishnan R. (2015). The role of MMP-2 and MMP-9 as prognostic markers in the early stages of tongue squamous cell carcinoma. *Journal of Oral Pathology & Medicine*.

[23] Määttä M., Soini Y., Liakka A., Autio-Harmainen H. (2000). Differential expression of matrix metalloproteinase (MMP)-2, MMP-9, and membrane type 1-MMP in hepatocellular and pancreatic adenocarcinoma: implications for tumor progression and clinical prognosis. *Clinical Cancer Research An Official Journal of the American Association for Cancer Research*.

[24] Ou Y., Li W., Li X., Lin Z., Li M. (2011). Sinomenine reduces invasion and migration ability in fibroblast-like synoviocytes cells co-cultured with activated human monocytic THP-1 cells by inhibiting the expression of MMP-2, MMP-9, CD147. *Rheumatology International*.

[25] Ning Y., Hui L., Wang Y., Yang H., Jiang X. (2014). SOX2 promotes the migration and invasion of laryngeal cancer cells by induction of MMP-2 via the PI3K/Akt/mTOR pathway. *Oncology Reports*.

[26] Esworthy R. S., Yang L., Frankel P. H., Chu F. F. (2005). Epithelium-specific glutathione peroxidase, Gpx2, is involved in the prevention of intestinal inflammation in selenium-deficient mice. *Journal of Nutrition*.

[27] Chen T., You Y., Jiang H., Wang Z. Z. (2017). Epithelial-mesenchymal transition (EMT): a biological process in the development, stem cell differentiation, and tumorigenesis. *Journal of Cellular Physiology*.

[28] Bao Y., Feng W. M., Tang C. W., Zheng Y. Y., Gong H. B., Hou E. G. (2012). Endostatin inhibits angiogenesis in hepatocellular carcinoma after transarterial chemoembolization. *Hepato-Gastroenterology*.

[29] Ulirsch J., Fan C., Knafl G. (2013). Vimentin DNA methylation predicts survival in breast cancer. *Breast Cancer Research and Treatment*.

[30] Yan P., Jing H., Zhang Y., Li X.-J. (2010). Role of vimentin in tumor metastasis and drug research. *Progress in Physiological Sciences*.

